# Left ventricular dyssynchrony in long-term childhood cancer survivors treated with anthracyclines: a retrospective cross-sectional study

**DOI:** 10.1007/s10554-021-02347-4

**Published:** 2021-08-06

**Authors:** Milanthy S. Pourier, Myrthe M. Dull, Gert Weijers, Jacqueline Loonen, Louise Bellersen, Chris L. de Korte, Livia Kapusta, Annelies M. C. Mavinkurve-Groothuis

**Affiliations:** 1 Department of Pediatrics, Amalia Children’s Hospital, Radboud University Medical Center, Nijmegen, The Netherlands; 2 Department of Radiology and Nuclear Medicine, Medical UltraSound Imaging Centre (MUSIC), Radboud University Medical Center, Nijmegen, The Netherlands; 3 Department of Hematology, Radboud University Medical Center, Nijmegen, The Netherlands; 4 Department of Cardiology, Radboud University Medical Center, Nijmegen, The Netherlands; 5 Pediatric Cardiology Unit, Tel-Aviv Sourasky Medical Center, Tel Aviv University, Sackler School of Medicine, Tel Aviv, Israel; 6 Department of Pediatric Cardiology, Amalia Children’s Hospital, Radboud University Medical Center, Nijmegen, The Netherlands; 7 Princess Máxima Center for Pediatric Oncology, Utrecht, The Netherlands

**Keywords:** 2D echocardiography, Cardiac toxicity, Myocardial strain, Strain rate imaging, Dyssynchrony

## Abstract

The purpose of this study was to investigate left ventricular contraction patterns in asymptomatic Childhood cancer survivors (CCS) using two-dimensional speckle tracking echocardiography (2DSTE). Left ventricular longitudinal and circumferential myocardial parameters were assessed using 2DSTE, in asymptomatic CCS and age matched healthy controls. Time to peak (T2P) systolic strain was quantified. Dyssynchrony index (DI) was measured by calculating the standard deviation of T2P systolic strain of six segments in each view. Difference between T2P systolic longitudinal strain of septal and lateral wall was also assessed as a parameter for dyssynchrony. We included 115 CCS with a median age of 17.2 years (range 5.6–39.5) and a median follow up of 11.3 years (range 4.9–29.5) and 119 controls. Conventional echocardiographic parameters and global longitudinal strain were significantly decreased in CCS compared to controls (p < 0.01 and p = 0.02, respectively). Dyssynchrony index did not differ between CCS and controls. There was a clinically insignificant smaller absolute difference between T2P systolic longitudinal of septal and lateral wall in CCS compared to controls. We showed no difference in longitudinal or circumferential left ventricular dyssynchrony in CCS compared to controls using 2DSTE. Future research should focus on assessing dyssynchrony in more segments and a larger CCS population, using both 2D and 3DSTE.

## Introduction

Long-term survival of children with cancer has improved over the last decades to approximately 70–80%. Anthracyclines are used in many cancer treatment protocols and have a known cardiotoxic effect. Cardiac disease is seen in 10.6% of Childhood cancer survivors (CCS) and increases to 27.8% if radiotherapy has been given [1]. Mechanisms of anthracycline-induced cardiotoxicity have been studied extensively. There is a role for cardiomyocyte apoptosis due to DNA damage by topoisomerase 2b and formation of reactive oxygen species [2]. However, cardiac extracellular matrix remodeling (cardiac fibrosis) may represent an additional important mechanism contributing to impaired Left ventricular (LV) function and adverse cardiac outcome [3–5]. This fibrosis is measurable by calculating extracellular volume fraction by cardiac magnetic resonance and has been found in CCS [6, 7]. Fibrosis has also been shown in myocardial biopsies from patients with anthracycline cardiomyopathy [8]. Fibrosis of the myocardial wall has been correlated to dyssynchronous myocardial contraction in patients after myocardial infarction and in patients with non-ischemic dilated cardiomyopathy [9, 10]. This may also occur in CCS. Currently, few studies assessed Left ventricular dyssynchrony (LVD) as a marker for anthracycline-induced cardiotoxicity. Strain-derived mechanical dyssynchrony abnormalities have been independently associated with Left ventricular ejection fraction (LVEF) changes over time in idiopathic dilated cardiomyopathy [11]. Decreased Global longitudinal strain (GLS) has already been used as an early sign of anthracycline-induced cardiotoxicity [12]. We hypothesize that increased left ventricular dyssynchrony could be an early sign of subclinical cardiotoxicity in asymptomatic CCS previously treated with anthracyclines. Differences in Time to peak (T2P) strain might be useful as a quick and easily accessible, bedside method to assess LVD. In this study, we aim to quantify T2P systolic longitudinal and circumferential strain and possible LVD in CCS using 2DSTE.

## Methods

### Study population

CCS who received anthracyclines as part of their cancer therapy, were included in this study when they visited our Late Effects outpatient clinic between December 2005 and November 2009. Exclusion criteria were: (1) Clinical heart failure, defined by the New York Heart Association (adults, NYHA, class II–IV), and the modified Ross heart failure classification (children), (2) history of cardiovascular disease or (3) chronic renal disease. The control group consisted of 119 healthy age-matched controls (children and adults), routinely referred for echocardiographic evaluation of an asymptomatic, innocent heart murmur or for screening purposes. Their medical history, electrocardiogram, and echocardiogram were not indicative of cardiac disease. For both groups, subjects were excluded when having poor echocardiographic image quality (framerate < 60/minute) or incomplete image acquisition. The study was approved by the local ethical committee and informed consent was obtained from all survivors (and their parents, when indicated).

### Echocardiographic image acquisition and analysis

A detailed transthoracic echocardiographic examination in left lateral position was performed according to the recommendations of the American Society of Echocardiography [13]. Images were obtained with a 3.0 MHz or a 5.0 MHz phased-array transducer, depending on age and weight of the subject, using a commercially available Vivid 7 echocardiographic scanner (GE, Vingmed Ultrasound, Horton, Norway). Quantification of cardiac chamber size, ventricular mass and LV function were done in accordance with American and European guidelines [13]. Left ventricular End-systolic wall stress (ESWS) was calculated using the modified formula of Rowland and Gutgesell [14]. Since the study group consisted of children and adults, left ventricular dimensions were indexed for Body surface area (BSA), not by using Z scores. Systolic function was determined using Left ventricular shortening fraction (LVSF) and LVEF using Biplane Simpson’s method. Left ventricular mass (LVM) was calculated using the formula of Devereux and Reichek [15].

Two-dimensional multi-frame B-mode images were obtained in apical 4-chamber view (4CH) for the Longitudinal strain (LS) and in parasternal mid-cavity short-axis view (papillary muscle level: SaxPM) for the Circumferential strain (CS), as described earlier by our group [16]. GLS, global systolic LS rate (GLSr), global systolic CS (GCS) and global CS rate (GCSr) were calculated, using the mean of three heart cycles. Global T2P systolic strain was calculated by averaging T2P systolic strain values of all segments for both views.

**Fig. 1:**
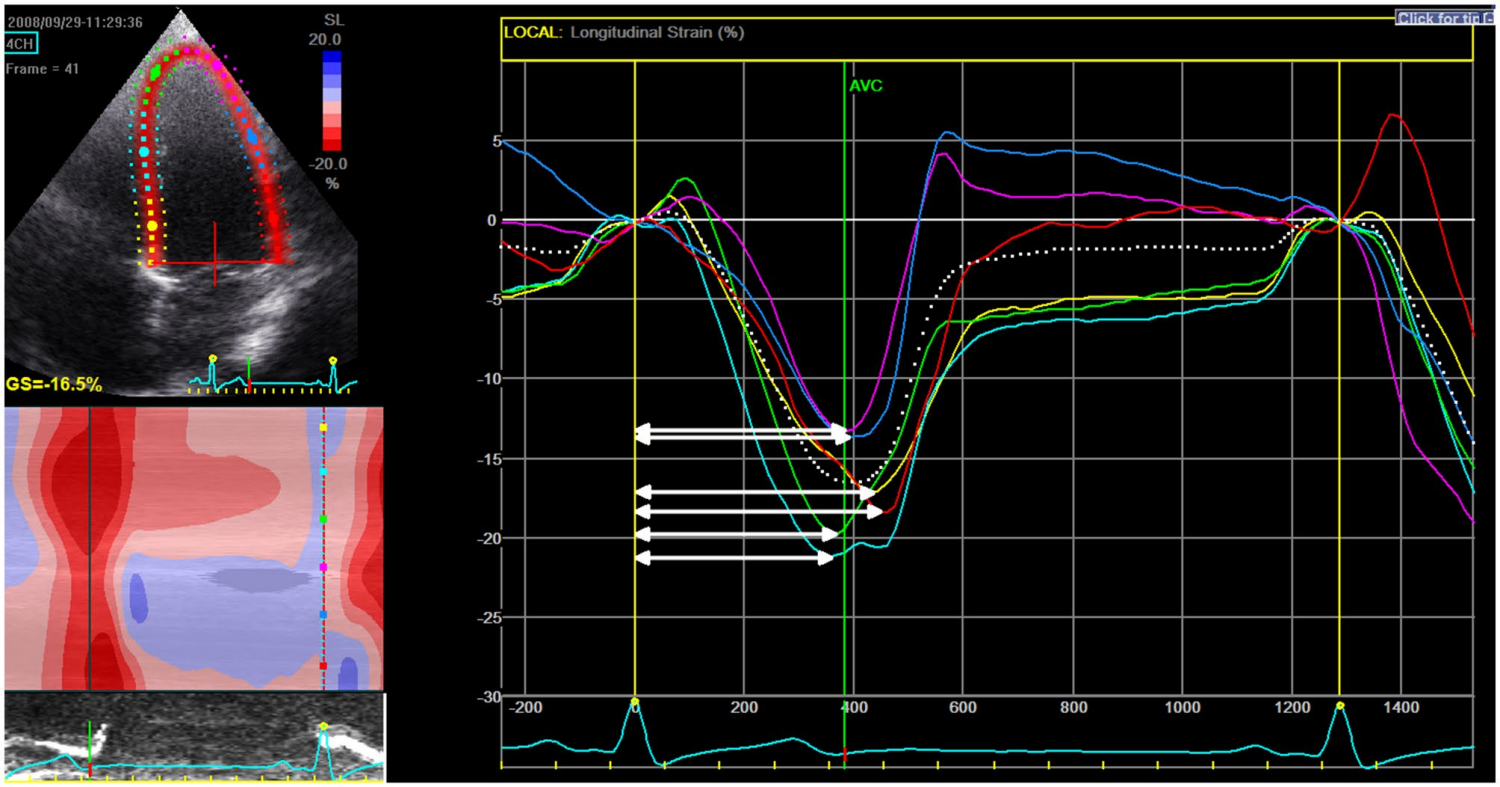
Measurement of dyssynchrony index in longitudinal strain. Calculation of dyssynchrony index (DI): Three cycles of longitudinal strain (LS) were measured. T2P systolic LS (indicated by the arrows) was calculated per segment (average of three cycles). DI was quantified by calculating the standard deviation of T2P systolic strain of six segments

To measure the extent of mechanical LVD, Dyssynchrony indices (DI), the Standard deviation (SD) of the T2P systolic strain in the longitudinal and circumferential direction were calculated (Fig. 1). In addition, difference between T2P systolic LS between septal and lateral wall was calculated in 4CH view [17]. Correction for the influence of heart rate on timing parameters was achieved by representing values as a percentage of the heart cycle.

### Inter- and intra-observer reproducibility

Inter-observer reproducibility of strain measurement was performed by two experienced analysts (M.P and A.M-G) in 10 CCS. Intra-observer reproducibility was performed by one analyst (M.P) repeating the same analysis in 10 CCS, blinded to previous measurements.

### Statistical analysis

Demographic and anthropometric values were summarized as median and range using a non-parametric test. Echocardiographic parameters were summarized as mean and SD. Characteristics of survivors and controls were compared using independent sample t-test. A two-tailed p-value of less than 0.05 was considered statistically significant. Correlations were studied using univariate analysis. Intraclass correlations (ICC) were calculated to assess intra- and interobserver variability using acknowledged cut-off points: < 0.20 poor agreement, 0.21–0.40 fair agreement, 0.41–0.60 a moderate agreement, 0.61–0.80 good agreement and 0.81–1.00 very good agreement. We considered an ICC > 0.60 as acceptable [18]. We used SPPS statistics, version 25 for statistical analysis.

## Results

We included 115 CCS and 119 healthy age matched controls in our study. None of the survivors met the exclusion criteria. Demographic characteristics are shown in Table 1. Except for a significantly lower blood pressure and a higher heart rate in the CCS group, characteristics were similar for both groups.

**Table 1:** Characteristics of study population

	Survivors	Controls	*P-value
	(n = 115)	(n = 119)	
Male	73 (63%)	77 (64%)	
Age at diagnosis	4.3 (0.03–16.9)		
Age at follow-up	17.2 (5.6–39.5)	16.6 (5.8–39.8)	0.79
< 18 years	60 (52%)	64 (54%)	
Follow-up duration after diagnosis (years)	11.3 (4.9–29.5)		
Body surface area (m^2^)	1.7 (0.8–2.3)	1.7 (0.8–2.4)	0.43
BMI (kg/m^2^)	20.3 (13.2–33.8)	20.6 (13.4–31.6)	0.60
Heart rate (bpm)	73 (47–107)	67 (43–115)	** < 0.01**
Mean arterial pressure (mmHg)	83 (60–116)	87 (62–105)	**0.03**
Height	1.7 (1.1–2.0)	1.69 (1.1–2.0)	1.00
Weight	60 (18–103)	60 (20–106)	0.59
Cumulative anthracycline dose (mg/m^2^) median (range)	180 (50–542)		
< 120	21		
120–300	62		
> 300	32		
Diagnosis			
Acute lymphoblastic leukemia (ALL)	36		
Acute myeloid leukemia (AML)	10		
Non Hodgkins lymphoma	21		
Hodgkins disease	10		
Neuroblastoma	9		
Wilms tumor	11		
Rhabdomyosarcoma	4		
Hepatoblastoma	5		
Osteosarcoma	3		
Ewing sarcoma	3		
Ependymoma	1		
Nasopharyngeal carcinoma	1		
Kidney tumor other than Wilms	1		

Values expressed as median and range

*Mann–Whitney-U test

Feasability of global T2P systolic LS and longitudinal DI was 99% in CCS and 97% in controls. Feasability of global T2P systolic CS and circumferential DI was 77% in CCS and 82% in controls. Feasability of difference in T2P systolic LS of septal and lateral wall was 96% in CCS and 94% in controls.

Table 2 presents conventional and 2DSTE parameters of both groups. Most conventional echocardiographic parameters were significantly decreased in CCS compared to controls. ESWS was significantly increased in CCS compared to controls. GLS was significantly decreased in CCS compared to controls.

**Table 2:** Conventional and myocardial 2D parameters in CCS and controls

	Survivors	Controls	P-value*
Number (N)	115	119	
Conventional parameters			
LVSF	35 ± 4	38 ± 5	** < 0.01**
LVEF	58 ± 8	68 ± 7	** < 0.01**
E/A ratio	2.06 ± 0.64	2.20 ± 0.74	0.20
ESWS	61.0 ± 18.1	50.3 ± 14.3	** < 0.01**
Left ventricular dimensions (cm/m^2^)			
LVIDd/BSA	3.16 ± 0.58	3.10 ± 0.62	0.57
LVIDs/BSA	2.03 ± 0.40	1.94 ± 0.44	0.12
LVPWd/BSA	0.41 ± 0.09	0.47 ± 0.09	** < 0.01**
LVPWs/BSA	0.72 ± 0.14	0.83 ± 0.17	** < 0.01**
IVSd/BSA	0.36 ± 0.09	0.43 ± 0.09	** < 0.01**
IVSs/BSA	0.57 ± 0.12	0.69 ± 0.19	** < 0.01**
LVM/BSA	57.14 ± 14.89	68.96 ± 18.86	** < 0.01**
Strain parameters			
GLS (%)	− 18.54 ± 2.53	− 19.37 ± 2.69	**0.02**
GLSr (1/s)	− 1.16 ± 0.19	− 1.16 ± 0.19	0.96
GCS (%)	− 19.48 ± 2.88	− 19.78 ± 2.86	0.48
GCSr (1/s)	− 1.53 ± 0.30	− 1.51 ± 0.30	0.79

Values expressed as mean ± SD

*BSA* body surface area, *E/A* ratio ratio early and late diastolic filling mitral valve, *ESWS* end systolic wall stress, *GCS* global circumferential strain, *GCSr* global circumferential strain rate, *GLS* global longitudinal strain, *GLSr* global longitudinal strain rate, *IVSd* diastolic intraventricular septum diameter, *IVSs* systolic intraventricular septum diameter, *LVEF* left ventricular ejection fraction, *LVIDd* diastolic left ventricle diameter, *LVIDs* systolic left ventricle diameter, *LVSF* left ventricular shortening fraction, *LVM* left ventricular mass, *LVPWd* diastolic left ventricular posterior wall, *LVPWs* diastolic left ventricular posterior wall

*P-value was calculated using independent sample t-test

Table 3 presents parameters of timing and LVD in survivors and controls. Global T2P systolic LS was shorter in CCS compared to controls. When corrected for heartrate this difference disappeared. Longitudinal DI (uncorrected for heartrate) was lower in CCS versus controls. This difference disappeared when corrected for heartrate. There was a significantly smaller absolute difference in global T2P systolic LS between septal and lateral wall in asymptomatic CCS compared to controls. Comparing children and adults separately did not alter the results. There was no correlation between the longitudinal or the circumferential DI and follow-up duration, cumulative anthracycline dose, gender or age at diagnosis (data not shown). None of the survivors or controls had an increased QRS interval indicative of left or right bundle branch block (data not shown).

**Table 3:** Timing- and dyssynchrony parameters in CCS and controls

	Survivors	Controls	P-value*
Number (N)	115	119	
Time to peak strain (msec)			
Global T2P systolic LS	338 ± 30	357 ± 29	** < 0.01**
Global T2P systolic CS	321 ± 30	333 ± 30	** < 0.01**
Time to peak (% heart cycle)			
Global T2P systolic LS	39 ± 6	39 ± 6	0.34
Global T2P systolic CS	38 ± 5	37 ± 6	0.46
Dyssynchrony index, DI (msec)			
4CH			
Longitudinal DI (uncorrected for HR)	26.1 ± 9.5	29.1 ± 10.4	**0.02**
Longitudinal DI (% heart cycle)	3.05 ± 1.21	3.18 ± 1.16	0.41
Diff SEPT/LAT (absolute value)	20.2 ± 15.2	26.2 ± 17.5	** < 0.01**
SaxPM			
Circumferential DI (uncorrected for HR)	32.0 ± 14.9	35.0 ± 18.8	0.23
Circumferential DI (% heart cycle)	3.74 ± 1.83	3.92 ± 2.13	0.53

Values expressed as mean ± SD

*DI* dyssynchrony index, *LS* longitudinal strain, CS circumferential strain, *Diff SEPT/LAT* difference between T2P systolic LS of septal and lateral wall, *HR* heart rate, *T2P* time to peak

*P-value was calculated using independent sample t-test

ICCs for dyssynchrony parameters for intra-observer and inter-observer reproducibility ranged from 0.67 to 0.83 and 0.68 to 0.94 respectively.

## Discussion

This pilot study assessed LV contracting patterns and the extent of LVD in CCS and healthy controls. Longitudinal DI, uncorrected for heart rate, was significantly lower in survivors compared to controls. We question the clinical relevance of this difference of 3 ms. When comparing our control values to a study by den Boer et al., the mean DI of the controls was similar (29 versus 30 ms). We found a broader range of DI in our control population compared to den Boer et al., possibly due to a larger age range in our population [20]. Klitsie et al. found an increasing DI with age in the pediatric population [19]. Our range of DI (children as well as adults) in survivors and controls fits their normal range of values supporting the conclusion that this small statistically significant difference in the longitudinal DI is clinically irrelevant. When longitudinal DI was corrected for heart rate, statistical significance disappeared. We found a smaller difference in global T2P systolic LS between septal and lateral wall in survivors compared to controls (22.3 versus 27.9 ms, p-value 0.019) which seems clinically irrelevant. Cut off values for LVD in predicting successful resynchronization therapy have been recently described by Mele et al. [21]. However, no cut-off values for LVD in predicting LV dysfunction in CCS have been identified.

To measure the DI in assessing LVD, different imaging techniques such as tissue Doppler (calculating the Yu-index), 2DSTE (as done in our study), 3D echocardiography or MRI can be used [20]. Dyssynchrony as a marker of *early-onset* anthracycline-induced cardiotoxicity has been demonstrated by Li et al. with an increased peak systolic dispersion measured by SD of T2P systolic LS of 18 segments (4CH, 3CH and 2CH) [22]. Dyssynchrony as a marker of *late-onset* anthracycline-induced cardiotoxicity has been shown by Okama et al. investigating 32 CCS and 12 controls. CCS were divided according to existence of diastolic LV regional Wall motion abnormalities (WMA). More dyssynchrony, defined as an increased LV Systolic dyssynchrony index (SDI) of the radial strain was seen in CCS with WMA, compared to controls and compared to CCS without WMA, with preserved LVEF. No difference in SDI was seen between controls and survivors without WMA [23]. A possible histopathological explanation for these findings is fibrosis leading to WMA as well as an increased SDI. Cheung et al. reported on 45 CCS using 3D echocardiography and measured SD of time to minimal volume of 16 segments (as % of cardiac cycle) with a median follow-up duration of 6.3 years (4.46% versus 3.80% with 16% dyssynchrony in CCS) [24]. A study by Yu et al. using the same parameter of dyssynchrony with a median follow up of 7.2 years also found increased dyssynchrony in asymptomatic CCS (7.8% versus 4.9%) [25]. Ylänen et al. performed 3D echocardiography and measured dyssynchrony by calculating the SD of time to reach minimum systolic volume in 12 and in 16 segments rather than using 2DSTE. Follow-up was done in 71 CCS of which eight also received radiotherapy. The longitudinal DI of 16 segments correlated negatively with LVEF. In linear regression a higher DI was correlated with lower LVEF and cardiac irradiation [26].

In contrast with our results, all above authors found dyssynchrony in CCS by using either 3D echocardiography or 2D echocardiography including more segments (2-, 3- and 4-chamber views). Strain as well as volume derived variables have been used in previous research. Insufficient correlation between 2 and 3D echocardiography in assessing dyssynchrony has been reported previously by other authors and could partly explain the difference in results [27, 28].

Myocardial fibrosis can be divided in focal (patchy) fibrosis and diffuse interstitial fibrosis [29]. Detecting patchy fibrosis is done by using Late gadolinium enhancement (LGE) imaging and has been described in studies on anthracycline-induced cardiotoxicity in *early*- as well as *late-onset* cardiotoxicity [29, 30]. Diffuse interstitial fibrosis appears to be a more widely reported phenomenon in anthracycline-induced cardiotoxicity and can be measured by quantifying extracellular volume fraction using T1 Mapping [31–33]. In more diffuse fibrosis, longitudinal and circumferential dyssynchrony measured with the rough measure of only six segments can be missed.

We found a shorter global T2P systolic LS (uncorrected for heartrate) in survivors compared to controls. We showed a significantly higher heart rate in survivors compared to controls (p = 001). A possible explanation for an increased heart rate might by an increased sympathetic stimulation after anthracycline exposure [34, 35]. Consequently, when corrected for heart rate, the difference in global T2P systolic LS between CCS and controls was diminished (p = 0.335).

### Study limitations

Our study has several limitations. First, our pilot study included 115 CCS. A larger population may show statistically significant differences in LVD parameters; however, its clinical relevance should be further investigated. Second, in this study we assessed dyssynchrony using 2DSTE as a quick and easily accessible, bedside method. A more complete assessment of cardiac function using more advanced techniques, e.g., 3D echocardiography or MRI, might be a more sensitive method. Third, we were limited by use of only six segments. Even though this makes our method quickly accessible in children, the use of more segments (e.g., 16 or 18 segments) could be more sensitive in detecting more subtle changes in left ventricular contraction patterns.

## Conclusion

In this study, we could not demonstrate LVD in asymptomatic CCS at late follow-up after exposure to anthracyclines when compared to healthy controls. More research needs to be done assessing possible dyssynchrony in a larger population of asymptomatic CCS. We advise using more longitudinal segments and different types of imaging (3D echocardiography and MRI) and compare the results with simultaneous quantification of fibrosis.

## Data Availability

Upon request.
